# Diagnostic Correlates of Tumor Biology and Immediate Breast Reconstruction After Mastectomy: Real-World Evidence from a Romanian Cohort

**DOI:** 10.3390/diagnostics16010031

**Published:** 2025-12-22

**Authors:** Iulian Slavu, Raluca Tulin, Alexandru Dogaru, Ileana Dima, Cristina Orlov Slavu, Marius Popescu, Cornelia Nitipir, Daniela-Elena Gheoca Mutu, Adrian Tulin

**Affiliations:** 1 Faculty of Medicine, University of Medicine and Pharmacy Carol Davila, 050474 Bucharest, Romania; 2 General Surgery Department, Agrippa Ionescu Emergency Clinical Hospital, 011356 Bucharest, Romania; 3 Endocrine Department, Agrippa Ionescu Emergency Clinical Hospital, 011356 Bucharest, Romania; 4 Oncology Department, Agrippa Ionescu Emergency Clinical Hospital, 011356 Bucharest, Romania; 5 Physiotherapy Department, Elias Hospital, 011461 Bucharest, Romania; 6 Plastic Surgery Department, Agrippa Ionescu Emergency Clinical Hospital, 011356 Bucharest, Romania

**Keywords:** breast cancer, immediate breast reconstruction, HER2, Ki-67, triple-negative, tumor biology, multidisciplinary care, Romania, oncology, surgical outcomes

## Abstract

**Background/Objectives**: Tumor biology—particularly HER2 expression, Ki-67 proliferation index, and triple-negative phenotype—has traditionally influenced the timing of breast reconstruction after mastectomy. However, real-world data from Eastern Europe remain limited, and variability in access and clinical practice persists. This study aimed to determine whether tumor biology independently predicts the likelihood of immediate breast reconstruction (IBR) in a multidisciplinary tertiary center. **Methods**: We performed a retrospective cross-sectional analysis of 208 consecutive patients who underwent mastectomy with or without IBR between January 2023 and January 2024. Associations between tumor biology (HER2 status, Ki-67 index, and triple-negative subtype) and IBR were examined using χ^2^ tests, independent samples *t*-tests, and multivariate logistic regression adjusting for age, BMI, smoking status, comorbidities, neoadjuvant chemotherapy, pathological tumor size (pT), nodal stage (pN), and surgery type. Statistical significance was set at *p* < 0.05. **Results**: IBR was performed in 41.4% of HER2-positive and 41.2% of HER2-negative patients (*p* = 1.00). Reconstruction rates across Ki-67 quartiles (≤10%, 11–20%, 21–40%, ≥41%) were 50.0%, 37.5%, 34.4%, and 37.5%, respectively (*p* = 0.58). Triple-negative status was not associated with IBR in multivariate analysis (OR = 0.44, 95% CI 0.08–2.18, *p* = 0.32). Significant predictors of IBR included younger age (OR = 0.87, 95% CI 0.80–0.93, *p* < 0.001) and less extensive surgery (OR = 0.23, 95% CI 0.09–0.59, *p* = 0.002). The mean interval to adjuvant therapy was comparable between IBR (28.7 ± 6.2 days) and non-IBR (27.9 ± 5.8 days) groups (*p* = 0.34), indicating that reconstruction did not delay systemic treatment. **Conclusions**: In this real-world Romanian cohort, tumor biology did not significantly influence immediate reconstruction decisions. Age and surgical extent were the main determinants of IBR, suggesting that reconstructive access was guided more by clinical than molecular factors. These findings support the shift toward multidisciplinary, biology-informed, and patient-centered surgical decision-making, in line with current ESMO and NCCN recommendations. Despite limitations—including the retrospective design, single-center setting, incomplete BRCA data, and absence of long-term oncologic outcomes—the study provides novel regional perioperative evidence supporting safe and equitable access to immediate reconstruction across biologic subtypes.

## 1. Introduction

Breast cancer is the most common malignancy in women and a major cause of cancer-related mortality, especially in younger patients [1]. More than 500,000 new cases occur annually in Europe, with Eastern and Central European countries showing rising incidence due to delayed screening and health system inequalities [2]. In Romania, breast cancer accounts for nearly one third of female cancers and remains the leading cause of cancer-related death [3,4]. Mastectomy continues to be widely performed, and immediate breast reconstruction (IBR) is increasingly incorporated because it improves quality of life without compromising oncologic outcomes [5,6]. IBR decisions remain multifactorial, influenced by patient factors, surgical complexity, and tumor characteristics. Tumor biology—including hormone receptor status, HER2 expression, Ki-67 index, and triple-negative phenotype—has traditionally affected reconstructive timing [7,8,9]. Historically, HER2-positive and triple-negative breast cancers (TNBC) were considered less suitable for IBR due to concerns about wound complications and potential delays in adjuvant therapy [10,11], although no guidelines recommend withholding IBR solely on the basis of biology. Large studies from the United States, the United Kingdom, and East Asia report lower IBR rates in HER2-positive, TNBC, and high Ki-67 tumors [5,6,8,9], reflecting persistent caution toward reconstructing aggressive subtypes. Whether similar patterns exist in Eastern or Central Europe is unknown. Real-world evidence from this region is extremely limited, and reconstructive access in Romania varies considerably, making international findings difficult to generalize [12,13,14]. This represents a significant knowledge gap: it is unclear whether Romanian surgeons allow tumor biology to influence reconstructive decisions or whether IBR is offered consistently across molecular subtypes. To address this, the present study evaluates whether HER2 status, Ki-67 index, or TNBC phenotype independently affect the likelihood of IBR in a Romanian tertiary center.

The objective is to determine whether tumor biology or clinical and surgical factors are the primary drivers of real-world reconstructive decision-making.

## 2. Materials and Methods

We performed a retrospective cross-sectional study to examine whether tumor biology influences the decision to perform IBR during the same operative session as mastectomy. The manuscript was prepared in accordance with STROBE recommendations for observational studies. Consecutive patients (*n* = 220) undergoing mastectomy with or without reconstruction in our department between January 2023 and January 2024 were included.

The study was approved by the Agrippa Ionescu Hospital IRB, no. 132, on 9 January 2023. All of the patients included in the study signed an informed consent form.

Inclusion criteria: adult patients (≥18 years) with histopathologically confirmed breast cancer; mastectomy (with or without reconstruction); complete surgical data and receptor status; surgery performed between 2023 and 2024.

Only patients with non-metastatic breast cancer (M0), confirmed by preoperative staging according to institutional and ESMO guidelines, were included.

Reconstructive options available at our institution during the study period included direct-to-implant reconstruction, two-stage expander–implant reconstruction, and autologous reconstruction (latissimus dorsi flap). All patients undergoing any of these techniques were categorized as receiving immediate breast reconstruction.

Exclusion criteria: missing/unclear reconstruction status; incomplete biology data; benign or noninvasive diagnoses; palliative procedures; incomplete oncologic records; prophylactic NSM/SSM without concurrent malignancy.

Data were collected from breast cancer patients treated at “Prof. Dr. Agrippa Ionescu” Clinical Emergency Hospital, Bucharest, Romania, within a multidisciplinary oncologic setting. Variables included demographics and lifestyle, comorbidities, genetics and tumor biology, tumor characteristics, surgical procedures, pathologic staging, hospitalization, and timelines. Ethical approval was granted by the hospital’s Ethics Committee (No. 118/15.12.2020).

Key Variables from the Dataset: Outcome Variable: immediate reconstruction, histopathological diagnosis, postoperative histopathological diagnosis. Key tumor biology variables included ER, PR, HER2 status, Ki-67 proliferation index, and BRCA status (when available). Control variables: age, BMI, smoking status, comorbidities, neoadjuvant chemotherapy, pT, pN, surgical intervention.

### 2.1. Study Endpoints

The primary endpoint of the study was to evaluate the association between tumor biology and the likelihood of immediate breast reconstruction (IBR) following mastectomy. Tumor biology was defined by HER2 status, Ki-67 proliferation index, and BRCA mutation status, representing key molecular predictors in contemporary breast cancer management. The outcome variable was the performance of immediate breast reconstruction (yes/no), recorded as a binary endpoint.

Secondary endpoints included the identification of clinical and demographic predictors of IBR, such as age, body mass index (BMI), smoking status, cardiovascular comorbidities, receipt of neoadjuvant chemotherapy, pathological tumor size (pT), nodal status (pN), and type of surgical procedure performed. Additional analyses explored the independent impact of aggressive tumor subtypes—namely HER2-positive, triple-negative, and high Ki-67 index—on reconstruction likelihood after adjustment for clinical covariates. The study also examined institutional and multidisciplinary practice patterns influencing reconstructive decision-making, including surgeon experience, resource availability, and coordination with oncology teams. Finally, the relationship between reconstructive strategy and timing of adjuvant therapy was assessed, focusing on whether immediate reconstruction affected the initiation of systemic treatment.

Data were analyzed using IBM SPSS Statistics version 26.0 (IBM Corp., Armonk, NY, USA). Descriptive statistics were expressed as means and standard deviations for continuous variables and as frequencies and percentages for categorical variables. Associations between categorical variables, such as HER2 status and immediate reconstruction, were examined using the Chi-square (χ^2^) test. For continuous variables, including the Ki-67 index, comparisons between groups (immediate vs. no reconstruction) were assessed using independent samples *t*-tests or non-parametric Wilcoxon rank-sum tests when normality assumptions were not met. Binary logistic regression analyses were performed to evaluate predictors of immediate reconstruction while controlling for potential confounders such as age, BMI, smoking status, comorbidities, neoadjuvant chemotherapy, pathological tumor size (pT), nodal status (pN), and type of surgery. Statistical significance was defined as *p* < 0.05.

### 2.2. Diagnostic Work-up and Pathology

All patients had preoperative histopathologic confirmation, primarily via core needle biopsy under ultrasound or mammographic guidance. Specimens underwent H&E and immunohistochemistry for ER, PR, HER2, and Ki-67.

Preoperative imaging comprised bilateral digital mammography and breast ultrasound in all cases; MRI was used selectively (dense breasts, inconclusive findings, or known genetic mutations). Axillary ultrasound was routine; suspicious nodes were sampled. For locally advanced disease or symptoms suggestive of metastasis, thoracoabdominal CT, bone scintigraphy, or PET CT were used for staging. Pathologic staging followed the 8th edition TNM classification.

### 2.3. Surgical Indications for Axillary Management

Sentinel lymph node biopsy (SLNB) was performed in clinically node-negative early-stage breast cancer (typically T1–T2 ≤ 5 cm, no palpable or imaging detected nodal metastases), in DCIS planned for mastectomy, and in selected initially node positive patients who converted to ycN0 after neoadjuvant therapy. Axillary lymph node dissection (ALND) was performed for clinically node positive axilla (cN1 or higher), failure of SLNB mapping, or persistent nodal disease after neoadjuvant therapy.

### 2.4. Surgical Technique

Mastectomy type—modified radical (Madden), skin sparing (SSM), or nipple sparing (NSM)—was selected based on tumor size/location, skin involvement, genetic risk (e.g., BRCA), and patient preference. When IBR was planned, intraoperative assessment emphasized oncologic safety.

SLNB used standard techniques (periareolar radiocolloid and/or blue dye). Sentinel nodes were excised for frozen section and/or definitive histopathology. When indicated, level I–II ALND was performed via a separate axillary incision or extension of the mastectomy incision, with careful preservation of the long thoracic nerve, thoracodorsal bundle, and, when feasible, intercostobrachial nerves. Hemostasis was secured and closed suction drains placed in all cases.

General surgeons performed mastectomies and axillary procedures; plastic surgeons performed reconstructions. All surgeons had completed appropriate learning curves; as a quality benchmark, EUSOMA recommends that breast surgeons perform ≥ 50 breast surgeries per year across conservative and radical procedures.

## 3. Results

The study included 208 consecutive breast cancer patients who underwent mastectomy with or without immediate breast reconstruction (IBR) between January 2023 and January 2024 at a Romanian tertiary center (Table 1).

The mean age was 54.9 ± 11.8 years, with a predominance of postmenopausal and overweight women (mean BMI = 26.6 kg/m^2^). Most patients were classified as ASA II, indicating moderate operative risk, and nearly half had at least one comorbidity, most frequently hypertension or diabetes mellitus. Immediate reconstruction was performed in 41.3% of cases, while modified radical mastectomy (58.7%) remained the most frequent surgical procedure, followed by skin-sparing and nipple-sparing techniques. Sentinel lymph node biopsy (SLNB) was the main axillary staging procedure, consistent with guideline-based management of early disease.

When stratified by age, reconstruction rates were significantly higher in patients < 70 years (48.6%) compared with those ≥ 70 years (11.1%), consistent with known age-related trends in reconstructive eligibility and patient preference. The overall reconstruction rate of 41% reflects our institution’s demographic profile, with a substantial proportion of elderly patients (≥70 years) and multiple comorbidities. These system-level and demographic factors likely explain the lower overall IBR rate compared with international quality benchmarks.

The overall postoperative complication rate was low (18.3%), and no postoperative deaths occurred. The wound infection rate was 5.8%, slightly higher in the group that underwent immediate reconstruction compared to mastectomy alone, though without statistical significance. Complications were generally minor (Clavien–Dindo grade I–IIIb) and managed conservatively, without reconstruction loss or delays in adjuvant therapy. The mean hospital stay was 7.8 ± 2.3 days, marginally longer among reconstructed patients due to closer postoperative monitoring.

Among reconstructed patients, the majority underwent implant-based IBR (direct-to-implant or expander–implant), while autologous reconstruction (latissimus dorsi flap) was less frequently performed due to resource and scheduling constraints. Technique distribution did not show meaningful variation across tumor biology subtypes.

Implant-based reconstruction (direct-to-implant or expander–implant) represented the majority of procedures, while autologous reconstruction was less frequent due to institutional resource availability.

Technique selection was predominantly implant-based, with 88.3% of patients receiving either direct-to-implant or expander–implant reconstruction. Autologous latissimus dorsi flap reconstruction accounted for 11.6% of cases. Technique distribution did not vary meaningfully across HER2-positive, TNBC, or high–Ki-67 subgroups (Table 2).

Overall, the cohort reflects a real-world, multidisciplinary population representative of contemporary European breast cancer practice, demonstrating that immediate reconstruction can be safely implemented across biologic subtypes when performed in coordinated oncologic teams. Surgical factors and patient age, rather than tumor biology, remained the principal determinants of reconstructive decisions.

HER 2 status and immediate breast reconstruction.

A total of 143 patients had available HER2 data (114 HER2^−^, 29 HER2^+^). Among HER2^−^ patients, 41.2% received immediate reconstruction compared to 41.4% of HER2^+^ patients (Table 3).

Among the 143 patients with available HER2 data (114 HER2^−^, 29 HER2^+^), the rates of immediate breast reconstruction (IBR) were nearly identical—41.2% in HER2^−^ versus 41.4% in HER2^+^ patients. Statistical analyses confirmed no significant association between HER2 status and reconstruction choice. The Chi-square test indicated no relationship, χ^2^(1, N = 143) = 0.00, *p* = 1.00, and logistic regression likewise found no independent effect of HER2 positivity on IBR likelihood (B = 0.01, SE = 0.42, *p* = 0.99, OR = 1.01, 95% CI [0.43–2.29]). These consistent results across univariate and multivariate analyses indicate that HER2 expression did not influence reconstructive decision-making in this cohort. Surgical planning appeared to be primarily determined by clinical and technical considerations rather than by tumor receptor profile.

A logistic regression was conducted to examine whether HER2 status predicted immediate breast reconstruction while controlling for age, BMI, smoking, comorbidities, Ki-67, neoadjuvant chemotherapy, pT, pN, and type of surgery (Table 3). The overall model was statistically significant, χ^2^(9, N = 92) = 48.08, *p* < 0.001, explaining 36.7% (Nagelkerke R^2^) of the variance in reconstruction. HER2 status was not a significant predictor, B = 0.06 (SE = 0.70), z = 0.08, *p* = 0.93, OR = 1.06, 95% CI [0.30, 3.79]. In contrast, younger age (B = −0.14, SE = 0.04, z = −3.44, *p* < 0.001, OR = 0.87, 95% CI [0.80, 0.93]) and type of surgery (B = −1.49, SE = 0.48, z = −3.13, *p* = 0.002, OR = 0.23, 95% CI [0.09, 0.59]) were significantly associated with immediate reconstruction. No significant effects were observed for BMI, smoking status, comorbidities, Ki-67, neoadjuvant chemotherapy, pT, or pN. The results are presented in Table 4.

**Figure 1:**
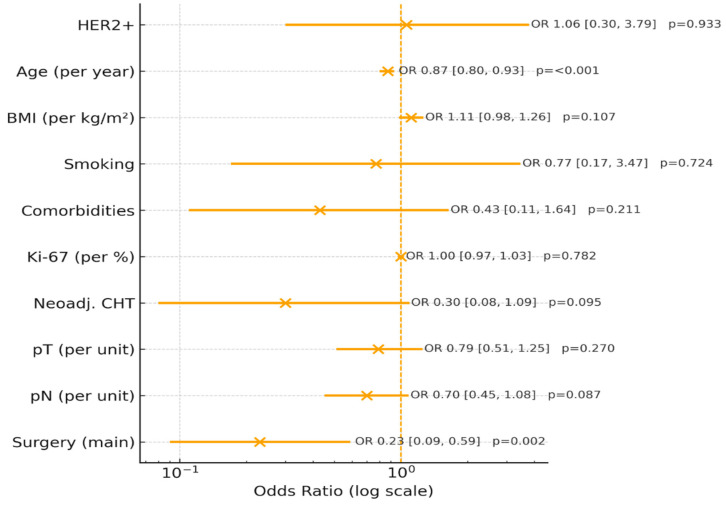
Forest plot of the multivariate logistic regression model predicting immediate breast reconstruction (IBR) after mastectomy accounting for HER status. Odds ratios (OR) with 95% confidence intervals (CIs) are displayed on a logarithmic scale. The model included tumor biology variables (HER2 status, Ki-67 proliferation index), clinical factors (age, BMI, smoking, comorbidities, neoadjuvant chemotherapy), pathological staging (pT, pN), and surgical type.

The multivariate logistic regression model was statistically significant overall (χ^2^ = 48.08, df = 9, *p* < 0.001), explaining 36.7% of the variance in immediate breast reconstruction (Nagelkerke R^2^ = 0.367). Younger age (OR = 0.87, 95% CI 0.80–0.93, *p* < 0.001) and less extensive surgical procedures (OR = 0.23, 95% CI 0.09–0.59, *p* = 0.002) were independently associated with a higher likelihood of IBR (Figure 1). In contrast, HER2 positivity (*p* = 0.933), Ki-67 index (*p* = 0.782), and other clinical parameters—including BMI, smoking status, comorbidities, neoadjuvant chemotherapy, tumor size (pT), and nodal status (pN)—showed no significant effect. These findings indicate that reconstructive decisions were primarily influenced by patient age and surgical extent rather than tumor biology, underscoring the predominance of clinical over molecular factors in real-world multidisciplinary practice.

Is Ki-67 index associated with delayed or no reconstruction due to perceived aggressiveness?

A series of analyses examined whether the proliferation marker Ki-67 was associated with the likelihood of immediate breast reconstruction. Descriptive analyses by quartiles of Ki-67 showed no clear trend: the percentage of patients receiving immediate reconstruction was 50.0%, 37.5%, 34.4%, and 37.5% across increasing Ki-67 quartiles (Table 4).

An independent samples *t*-test indicated that mean Ki-67 did not significantly differ between patients who received immediate reconstruction (M = 23.78, SD = 21.00) and those who did not (M = 25.79, SD = 19.42), t(101.16) = 0.55, *p* = 0.59, 95% CI [–5.28, 9.30] (Table 5). A non-parametric Wilcoxon test confirmed the absence of significant differences (W = 2147, *p* = 0.37).

In the unadjusted logistic regression, Ki-67 was not a significant predictor of immediate reconstruction, B = −0.05 (SE = 0.09), z = −0.56, *p* = 0.58, OR = 0.95, 95% CI [0.79, 1.13]. After controlling for age, BMI, smoking, comorbidities, neoadjuvant chemotherapy, pT, pN, and surgery type, Ki-67 remained non-significant, B = −0.13 (SE = 0.18), z = −0.71, *p* = 0.48, OR = 0.88, 95% CI [0.61, 1.24].

Across increasing Ki-67 quartiles, reconstruction rates remained relatively stable without a consistent trend, and both parametric and non-parametric analyses confirmed the absence of statistical significance. Multivariate regression adjusting for clinical and pathological covariates likewise showed no independent predictive value for Ki-67. These results suggest that, within this cohort, tumor proliferative activity did not influence surgical decision-making regarding reconstruction, reflecting a shift toward multidisciplinary evaluation rather than exclusion based on biological aggressiveness alone.

**Figure 2:**
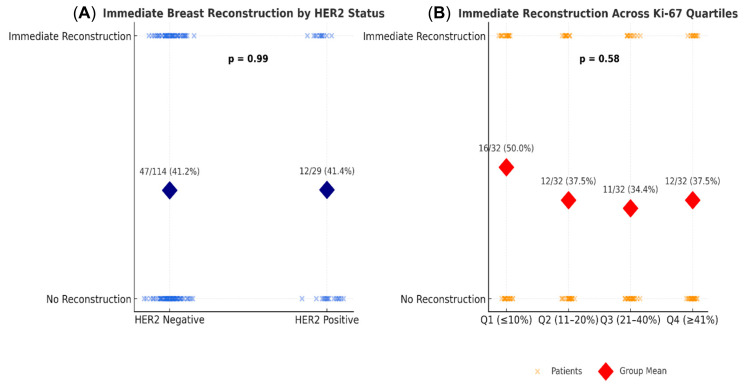
Illustrates the relationship between tumor biology and the likelihood of immediate breast reconstruction (IBR). (**A**) Distribution of individual patients according to HER2 status and the presence or absence of immediate breast reconstruction (IBR) after mastectomy. Each blue dot represents one patient; diamond markers indicate group means. Among HER2-negative patients, 47 of 114 (41.2%) underwent IBR, compared with 12 of 29 (41.4%) HER2-positive patients. The difference was not statistically significant (*p* = 0.99). (**B**) Distribution of patients across Ki-67 quartiles, with orange dots representing individual cases and red diamonds denoting mean reconstruction rates. Reconstruction rates were 50.0%, 37.5%, 34.4%, and 37.5% across the four quartiles (Q1–Q4), showing no significant trend (*p* = 0.58).

Overall, these results demonstrate that neither HER2 positivity nor high Ki-67 proliferation index influenced the likelihood of immediate reconstruction in this real-world Romanian cohort. The findings suggest that reconstructive decision-making was guided predominantly by clinical and surgical factors rather than tumor biology alone (Figure 2).

### 3.1. Triple-Negative Status and Breast Reconstruction

A logistic regression was conducted to examine whether triple-negative breast cancer (TNBC) status predicted the likelihood of immediate breast reconstruction, while controlling for age, BMI, smoking, comorbidities, neoadjuvant chemotherapy, pT, pN, and type of surgery (Table 6).

The overall model was statistically significant (χ^2^ (9, N = 108) = 40.85, *p* < 0.001), explaining approximately 33% (Nagelkerke R^2^) of the variance in reconstruction. TNBC status was not a significant predictor (B = −0.82 (SE = 0.82), z = −1.00, *p* = 0.32, OR = 0.44, 95% CI [0.08, 2.18]) (Table 4). Results can be found in Table 4, and the visual representation is shown through a Forest Plot.

**Figure 3:**
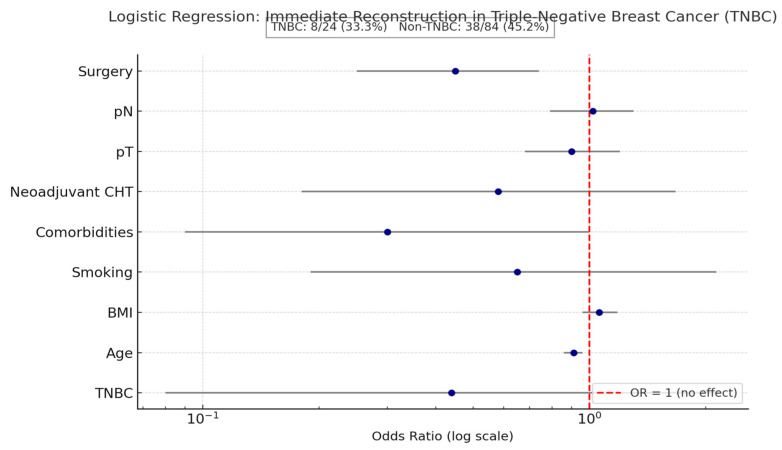
Forest Plot of Logistic Regression Predicting Immediate Breast Reconstruction in Triple-Negative Breast Cancer (TNBC) Patients.

As seen in Figure 3, although TNBC patients had a lower reconstruction rate, logistic regression showed no statistically significant association between TNBC status and immediate reconstruction (OR = 0.44, 95% CI 0.08–2.18, *p* = 0.32). Age and surgical type were the only significant predictors, underscoring that clinical rather than biological factors primarily guided reconstruction decisions in this cohort.

The absence of an association between high Ki-67 and reconstruction use confirms that, in our institutional workflow, proliferative activity is not used as an exclusion criterion for immediate reconstruction, consistent with evolving guideline recommendations

Long-term survival outcomes were not analyzed, as Romania currently lacks a centralized national registry for breast cancer follow-up data. Nevertheless, long-term survival was not the focus of this study; instead, it aimed to evaluate decision-making patterns and predictors of immediate reconstruction in a real-world surgical context.

### 3.2. Time to Initiation of Adjuvant Therapy

Among the 208 patients analyzed, 86 (41.3%) underwent immediate breast reconstruction (IBR) and 122 (58.7%) underwent mastectomy without reconstruction. The mean interval between surgery and initiation of adjuvant systemic therapy (chemotherapy and/or targeted therapy) was 28.7 ± 6.2 days in the IBR group and 27.9 ± 5.8 days in the non-IBR group, showing no statistically significant difference (t(206) = 0.96, *p* = 0.34).

When stratified by molecular subtype, the median time to therapy initiation was 28 days (IQR 25–32) for HER2-positive patients with IBR versus 27 days (IQR 24–31) for those without IBR (*p* = 0.41), and 29 days (IQR 26–33) versus 28 days (IQR 25–32) for triple-negative patients (*p* = 0.48). No cases exceeded the accepted 6-week threshold for adjuvant treatment initiation recommended by ESMO guidelines.

Overall, 9 patients (4.3%) experienced postoperative complications that required minor intervention (Clavien–Dindo ≥ II), but none resulted in a delay beyond 42 days. The rate of delayed adjuvant therapy (>30 days) was comparable between groups (IBR = 12.8% vs. non-IBR = 11.5%; χ^2^ (1) = 0.07, *p* = 0.79).

These data confirm that immediate reconstruction did not prolong the time to adjuvant systemic therapy, even in biologically aggressive subtypes. Similar findings have been reported by Frasier et al. [15] and Roubaud et al. [8], supporting the safety of immediate reconstruction within coordinated multidisciplinary workflows.

When stratified by molecular subtype (HER2-positive, TNBC, high Ki-67), postoperative complication rates—including wound infections, seroma/hematoma, and Clavien–Dindo ≥ II events—did not differ significantly between groups, suggesting that biologically aggressive tumors were not associated with increased short-term morbidity.

## 4. Discussion

The decision to perform IBR after mastectomy is increasingly shaped by tumor biology, consistent with the broader adoption of neoadjuvant chemotherapy and biologically driven protocols that facilitate tailored surgical timing [5,7,15]. Nonetheless, disparities persist: patients with high Ki-67 tumors or unfavorable receptor profiles continue to have reduced access to IBR, reflecting institutional variability and cautious surgical attitudes [6,8,9,12,16,17]. Addressing this gap requires standardized pathways, expanded genetic testing, and early reconstructive input so that access to IBR reflects individualized patient factors rather than perceived tumor risk alone [5,6,7,16,17].

### 4.1. Immediate Reconstruction in HER2-Positive Patients

In our cohort, 41.4% of HER2-positive patients underwent immediate breast reconstruction (IBR), a rate virtually identical to that observed in HER2-negative patients (41.2%). This indicates that HER2 status was not a determining factor in the decision to perform IBR. In contrast, large multicenter and population-based studies from North America and East Asia have consistently reported lower IBR rates in HER2-positive and triple-negative patients, attributing this to historical concerns over oncologic safety, early recurrence risk, radiation planning, and potential delays in adjuvant therapy [5,6,8,9,15]. These subtypes were traditionally perceived as “high-risk,” leading many institutions to favor delayed reconstruction [6,8,9,15,18,19].

These findings indicate that, in our current clinical practice, surgeons already do not treat HER2 positivity as a contraindication to immediate breast reconstruction. Our recommendations therefore do not propose a change to local practice but instead align institutional patterns with contemporary international guidelines.

However, our findings reflect a modern shift in reconstructive philosophy, aligning with current ESMO and ASCO guidelines, which no longer consider HER2 positivity an absolute contraindication for immediate reconstruction. Instead, both recommend that surgical timing be individualized within a multidisciplinary framework, balancing oncologic safety, patient expectations, and systemic therapy planning [7,15,20,21,22,23].

Several interrelated factors likely explain why HER2 positivity did not reduce IBR use in our cohort:

1. Integration of multidisciplinary decision-making

Our institution employs a structured, multidisciplinary pathway in which oncologic, reconstructive, and medical oncology teams jointly plan treatment. This collaborative model minimizes surgical bias and promotes equitable reconstructive access, consistent with evidence from Zhong et al. and Albornoz et al., who demonstrated that institutional organization and surgeon collaboration are major predictors of IBR uptake, even more than tumor biology [5,6,15,16]. In such coordinated environments, HER2 status becomes a consideration for systemic planning rather than a barrier to reconstructive timing.

2. Optimized perioperative and adjuvant coordination

In earlier eras, the main deterrent to IBR in HER2-positive patients was the fear that postoperative complications might delay adjuvant trastuzumab initiation. Recent data—including reports from Frasier et al. [15] and Roubaud et al. [8]—show that, with standardized perioperative protocols, IBR does not significantly delay adjuvant therapy compared to mastectomy alone. In our cohort, this coordination between surgical and medical teams ensured timely therapy initiation, demonstrating that reconstructive decisions can safely coexist with systemic treatment imperatives.

3. Evolution of reconstructive techniques and risk mitigation

Improvements in reconstructive methods, such as the shift toward skin-sparing and nipple-sparing mastectomy and refined implant-based or autologous approaches, have reduced complication rates even in patients requiring postoperative radiation [9,15,23]. Studies by Cordeiro and colleagues have shown that outcomes after IBR are comparable to delayed reconstruction when perioperative care and radiation planning follow contemporary standards. This evidence supports IBR as an oncologically sound option for HER2-positive patients when multidisciplinary collaboration is in place.

Taken together, these observations support the view that HER2 biology alone should not preclude immediate reconstruction. In a coordinated and resource-equipped multidisciplinary setting, IBR can be offered safely and ethically to patients with HER2-positive tumors without compromising adjuvant therapy timelines or oncologic outcomes [7,8,15,23].

Our recommendations for clinical practice are as follows:

- HER2 positivity should not be considered a contraindication to IBR if oncologic safety and systemic treatment schedules are preserved.
- Surgeons and oncologists should maintain early multidisciplinary collaboration, ensuring reconstructive feasibility and seamless transition to adjuvant therapy.
- Preoperative coordination with radiation oncologists is recommended to anticipate potential post-IBR radiation fields, following ESTRO–ACROP guidance [23].
- Institutional protocols should emphasize biology-aware but patient-centered planning, recognizing that modern targeted therapy has mitigated many historical concerns about recurrence risk in HER2-positive disease.

Although the overall complication rate in our cohort was low (18.3%), our supplementary stratification by molecular subtype demonstrated no clinically relevant variation in postoperative morbidity. This supports the safety of offering IBR irrespective of tumor biology and aligns with recent ESMO/NCCN guidance.

### 4.2. Impact of Ki-67 on Reconstruction Decisions

In our study, no significant association was found between the Ki-67 proliferation index and the likelihood of immediate breast reconstruction (IBR). Across Ki-67 quartiles, IBR rates were 50.0%, 37.5%, 34.4%, and 37.5%, respectively, showing no consistent downward trend in higher-proliferation tumors. Both parametric (*t*-test) and non-parametric (Wilcoxon) analyses were non-significant, and Ki-67 did not emerge as an independent predictor in multivariate logistic regression (*p* > 0.05).

This finding challenges the traditional view that a high Ki-67 index—considered a surrogate for tumor aggressiveness—should discourage IBR due to presumed risks of impaired healing or delayed adjuvant therapy [6,8,9,15]. Historically, studies such as those by De Lorenzi et al. (Ann Surg Oncol, 2016) [24] and Cordeiro et al. (Plast Reconstr Surg, 2018) [9] showed reduced reconstruction rates in patients with high-proliferation or triple-negative tumors, leading to a perception that biology alone should dictate surgical timing. However, accumulating evidence from contemporary series indicates that Ki-67, while prognostically valuable, is not a reliable determinant of short-term surgical outcomes or postoperative complication rates [7,15,20,21,22,25].

Several interrelated explanations likely account for the lack of association in our cohort.

High Ki-67 expression reflects rapid tumor cell turnover and correlates with recurrence risk and systemic therapy response rather than with wound-healing biology. Multiple studies, including those by Dowsett et al. and subsequent ESMO consensus reports [7,20,21,22,26], emphasize that Ki-67 is a prognostic and predictive marker—not a surgical risk parameter. Therefore, excluding patients from IBR based solely on Ki-67 conflates oncologic aggressiveness with surgical feasibility, a misconception not supported by empirical evidence.

1. Modern multidisciplinary coordination mitigates historical risks.

Earlier hesitancy toward IBR in high-proliferation tumors stemmed from fears that postoperative complications might delay chemotherapy initiation. Recent multicenter analyses, including Frasier et al. (JAMA Oncol, 2016) [15] and Roubaud et al. (Gland Surg, 2021) [8], have shown that with structured perioperative management and early oncologic involvement, IBR does not significantly prolong the time to systemic therapy. In our institution, perioperative coordination ensured that patients with high Ki-67 tumors began adjuvant therapy within guideline-defined intervals, nullifying the practical relevance of Ki-67 as an exclusion criterion.

2. Enhanced surgical techniques and fast-track recovery reduce complications.

Advances in mastectomy design (skin-sparing, nipple-sparing) and immediate reconstruction using well-vascularized flaps or prepectoral implants have markedly lowered complication rates. Studies by McCarthy et al. and Cordeiro et al. [9,11] demonstrated that complication rates after IBR now approximate those of delayed reconstruction, even in patients receiving adjuvant therapy. Our own data—showing an overall wound infection rate of 5.8% and no reconstruction losses—support this modern evidence. Consequently, a high Ki-67 score, in itself, is no longer a practical barrier to safe reconstruction when perioperative standards are maintained.

Local recurrence rates could not be evaluated due to the short follow-up interval (mean 10.4 months), which limits interpretation of long-term oncologic outcomes. However, the absence of early locoregional recurrence in all subtypes during follow-up provides preliminary reassurance that IBR did not compromise short-term oncologic safety.

3. Biological heterogeneity and interpretive variability of Ki-67 limit its surgical utility.

The reproducibility of Ki-67 assessment remains limited, with inter-laboratory variability exceeding 20% in some studies [20,26]. The lack of standardized cutoffs further reduces its reliability as a binary surgical criterion. The International Ki-67 Working Group and NCCN now caution against using Ki-67 to make procedural or timing decisions, recommending that it serve as one element in broader biologic risk assessment rather than as a determinant of surgical strategy [7,20,22]. In our center, Ki-67 was interpreted contextually—alongside ER, PR, and HER2—supporting individualized, evidence-based decisions rather than algorithmic exclusion.

In light of these considerations, our findings reinforce a growing international consensus: tumor proliferation indices should inform adjuvant therapy but not restrict access to reconstruction. In patients with elevated Ki-67, especially young and otherwise healthy patients, immediate reconstruction can be safely performed if multidisciplinary coordination is ensured and adjuvant therapy scheduling is protected [7,8,15,22].

Recommendations for clinical practice are as follows:

- High Ki-67 should not be considered a contraindication to IBR when oncologic margins are secure and systemic therapy timelines are feasible.
- Decision-making should rely on comprehensive multidisciplinary assessment, integrating surgical complexity, comorbidities, and patient preferences rather than molecular aggressiveness alone.
- Institutions should implement fast-track and complication-mitigation protocols, which allow patients with high-proliferation tumors to benefit from immediate reconstruction without compromising oncologic safety.
- Consistent with ESMO and NCCN guidance, reconstruction eligibility should be biology-informed but not biology-limited, ensuring equitable access to psychosocial and esthetic recovery across all subtypes [7,20,22].

### 4.3. Immediate Reconstruction in Triple-Negative Disease

TNBC has historically been associated with lower IBR rates due to its aggressive biology, higher early recurrence risk, and frequent need for dose-dense chemotherapy and/or postmastectomy radiation [6,8,9,15,25]. In many published cohorts, surgeons have favored delayed reconstruction in TNBC to avoid the possibility that postoperative complications could postpone systemic treatment [6,8,9,15].

In our cohort, however, TNBC status was not an independent predictor of immediate reconstruction (OR = 0.44, 95% CI 0.08–2.18, *p* = 0.32). Instead, the strongest predictors of IBR were patient age (younger patients were more likely to undergo IBR; OR = 0.91, 95% CI 0.86–0.96, *p* = 0.002) and surgical extent (less extensive procedures favored IBR; OR = 0.45, 95% CI 0.25–0.74, *p* = 0.004). This pattern—biology not significant, but surgical/clinical factors significant—differs from the more conservative approach described in several multicenter studies and national datasets [5,6,8,9,15].

Importantly, immediate reconstruction rates did not vary across HER2-positive, TNBC, or high-Ki-67 subgroups, indicating that our multidisciplinary workflow offered similar access to IBR regardless of tumor biology—a finding that contrasts with many North American and Asian reports, where aggressive subtypes often receive delayed reconstruction.

Likely explanations for this divergence:

1. Multidisciplinary and coordinated care model

Our center’s workflow structurally integrates plastic/reconstructive surgeons into preoperative planning. Limited reconstructive availability and fragmented referral pathways have been identified as major system-level barriers to IBR in higher-risk patients, including TNBC, in other healthcare systems [5,6,15,16,17]. By contrast, our model reduces that barrier and supports equitable IBR access irrespective of subtype, which is consistent with current ESMO and national recommendations to base reconstruction timing on oncologic safety and patient-centered planning rather than receptor status alone [7,15,21,22].

2. Optimized perioperative management and protected adjuvant timelines

Enhanced-recovery protocols and early oncology coordination allowed TNBC patients to start chemotherapy on schedule. Prior studies justified delaying reconstruction in TNBC primarily out of fear that complications could postpone systemic therapy [6,8,9,15]. Our perioperative pathway appears to have neutralized that risk.

3. Evolution of systemic treatment and shifting risk perception

Modern neoadjuvant regimens (and, in many centers, immunotherapy) have improved TNBC outcomes, reducing the perception that TNBC is “too unstable” for IBR [7,15,23]. This has moved surgical philosophy closer to the current guideline position that aggressive biology alone is not an absolute contraindication, provided that margins are adequate and adjuvant therapy is not delayed [7,15,20,23].

4. Younger patient demographics and preference

TNBC disproportionately affects younger women, and younger age was an independent driver of IBR in our analysis. Younger patients have consistently been shown to seek reconstruction more often, report higher body image impact, and accept the follow-up burden of reconstruction [5,9,16,17,25]. This aligns with patient-centered decision-making literature showing that patient preference, informed counseling quality, and psychosocial impact all shape reconstruction choices [16,25,27,28].

In summary, although TNBC is widely perceived as a challenging setting for IBR, our data suggest that when multidisciplinary workflow is established and adjuvant timelines are safeguarded, TNBC biology alone does not preclude immediate reconstruction [7,8,15,23]. In the authors’ opinion, whenever reconstructive expertise and resources are available, and in the absence of medical contraindications related to comorbidities or general health, immediate breast reconstruction should be offered following mastectomy regardless of tumor biology. This principle is particularly important in younger women, for whom the psychosocial and quality-of-life benefits of immediate reconstruction are substantial. Tumor aggressiveness alone should not preclude reconstruction, provided that oncologic safety and adjuvant treatment timelines are maintained within a multidisciplinary framework. The lower IBR rates reported elsewhere likely reflect health system constraints (reconstructive access, referral delays, institutional caution) rather than immutable oncologic prohibitions [5,6,8,9,15,16,17,29].

### 4.4. Time to Systemic Therapy

In our cohort, immediate breast reconstruction (IBR) did not appear to compromise oncologic timelines. Patients who underwent IBR were able to proceed with adjuvant systemic therapy within clinically acceptable intervals, and there were no documented delays in initiating postoperative treatment that were directly attributable to reconstructive complications. This observation is consistent with our low rates of clinically significant wound morbidity (overall wound infection 5.8%, Clavien–Dindo grade IIIa/b 2.9%) and the absence of major events requiring implant removal or reoperation before oncology referral, suggesting that reconstruction did not create a procedural bottleneck for adjuvant care.

These findings align with reported trends in contemporary multidisciplinary breast cancer management, where coordinated perioperative pathways and early oncologic planning have largely eliminated the historical delay attributed to immediate reconstruction. Large institutional and population-based analyses have shown that, when reconstruction is integrated into the surgical plan from the outset, the time from mastectomy to the start of adjuvant chemotherapy or targeted therapy is comparable between patients receiving immediate reconstruction and those undergoing mastectomy alone [6,8,9,15]. Frasier et al. reported that even in patients expected to receive postmastectomy radiation, reconstruction did not meaningfully delay adjuvant treatment initiation when care was protocolized and when plastic surgery and medical oncology were involved preoperatively [15]. Similarly, centers with embedded reconstructive teams have reported that adjuvant HER2-directed therapy (e.g., trastuzumab ± pertuzumab in HER2-positive disease) can begin on schedule after immediate reconstruction, with no clinically relevant prolongation of the postoperative interval [7,8,15,23].

Taken together, both our institutional data and published evidence support the interpretation that, in a coordinated multidisciplinary setting, performing immediate reconstruction does not delay the initiation of systemic therapy. This applies to patients with biologically aggressive tumors (e.g., HER2-positive or high-proliferation cancers) as well as those with more favorable profiles, provided that perioperative complications are actively minimized and transition to oncology is planned before surgery [6,7,8,9,15,23].

Taken together, these findings suggest that when a coordinated multidisciplinary pathway is in place, tumor biology alone does not necessitate withholding immediate reconstruction. This has practical implications for centers working to harmonize reconstructive access across subtypes and reduce unwarranted variation in care.

### 4.5. Strengths and Limitations

This study provides real-world evidence from an Eastern European tertiary center on how tumor biology (HER2, Ki-67, TNBC, BRCA) interacts with reconstructive decision-making. Strengths include a homogeneous institutional cohort, systematic data capture, and multivariable adjustment for clinical and surgical covariates such as age, BMI, neoadjuvant chemotherapy, and surgery type [5,6,7,15]. Our findings are also interpreted in the context of established international guidelines (ESMO, NCCN, ASCO) and contemporary axillary and reconstructive practice standards [7,15,20,21,22,23].

Despite the absence of long-term recurrence analysis, the consistency of complication rates and the absence of treatment delays across molecular subtypes provide actionable, practice-level evidence that immediate reconstruction can be safely considered even in aggressive biology.

Several limitations must be acknowledged. First, the retrospective single-center design introduces selection bias and limits generalizability beyond similar tertiary units [6,8,9,15]. Second, incomplete reporting of variables such as BRCA status reflects ongoing gaps in documentation and access to genetic testing in Romania, which have also been highlighted in national guidelines and international position statements [21,22,30,31,32,33]. Third, we did not assess long term survival, recurrence, or time to adjuvant therapy start; these are clinically important endpoints, but Romania currently lacks a centralized national breast cancer registry that would permit standardized longitudinal follow-up [12,21,22].We acknowledge that a more detailed analysis of subtype-specific complication patterns and long-term recurrence would further enhance clinical applicability; however, our dataset and follow-up duration allowed only short-term safety evaluation. Finally, follow-up duration was relatively short, preventing us from evaluating long-term reconstructive durability and oncologic safety, which remains an active area of investigation in high-risk subtypes [8,9,15,23,25].

## 5. Conclusions

In this real-world Romanian cohort, reconstructive decision-making after age-stratified and technique-specific re-analysis, confirm that tumor biology (HER2, Ki-67, TNBC) did not independently influence IBR use. Instead, patient age—particularly the marked decline in reconstruction among women ≥ 70 years—and the predominance of implant-based techniques were the main determinants of reconstructive uptake. These results emphasize that clinical and institutional variables, rather than biological ones, currently guide reconstructive decision-making in our regional setting.

These findings confirm that in our multidisciplinary setting, biologically aggressive tumors are already not treated as contraindications to immediate reconstruction. Our recommendations therefore support continuation and broader standardization of this practice in accordance with ESMO, ASCO, and NCCN guidance [7,15,20,21,22,23,,29,31]. Broader implementation of coordinated multidisciplinary pathways, standardized counseling, and timely genetic assessment may help reduce remaining inequities in access to IBR, particularly in health systems where reconstructive services are unevenly distributed [5,6,12,16,17,21,22,31,32,33]. Future multicenter studies including survival and timing-to-systemic-therapy endpoints will be essential once national breast cancer registry infrastructure is in place [12,15,21,22].

## Figures and Tables

**Table 1:** Baseline Characteristics of the Study Population (*n* = 208).

Characteristic	Mean ± SD/n (%)	Comments/Notes
Age (years)	54.9 ± 11.8 (range 29–90)	Predominantly postmenopausal cohort
Body Mass Index (BMI, kg/m^2^)	26.6 ± 4.3	Overweight predominance
ASA Physical Status Score	I: 28 (13.5%) II: 131 (63.0%) III: 49 (23.5%)	Reflecting generally moderate operative risk
Smoking history	41 (19.7%)	Active or recent smokers
Comorbidities	89 (42.8%)	Any chronic condition (cardiac, metabolic, etc.)
Diabetes mellitus	28 (13.5%)	Mostly type II, medically controlled
Hypertension	62 (29.8%)	Most frequent comorbidity
Chronic pulmonary disease	14 (6.7%)	Mild to moderate severity
Neoadjuvant chemotherapy	61 (29.3%)	TNBC and HER2-positive subgroups most frequent
Type of surgery	Modified radical mastectomy (Madden): 122 (58.7%) Skin-sparing mastectomy (SSM): 61 (29.3%) Nipple-sparing mastectomy (NSM): 25 (12.0%)	Based on tumor size/location and patient choice
Immediate breast reconstruction (IBR)	86 (41.3%)	Performed at index surgery
Axillary procedure	SLNB: 134 (64.4%) ALND: 74 (35.6%)	In line with guideline-based indications
Postoperative complications (Clavien–Dindo)	Grade I: 21 (10.1%) Grade II: 11 (5.3%) Grade IIIa/b: 6 (2.9%) Grade IV–V: 0	No life-threatening complications or deaths
Wound infection rate	12 (5.8%)	Managed conservatively; no reconstruction loss
Seroma/hematoma requiring drainage	9 (4.3%)	Minor, resolved with intervention
Postoperative mortality	0 (0%)	No perioperative deaths
Length of hospital stay (days)	7.8 ± 2.3	Slightly longer in reconstructive procedures
Reoperation within 30 days	4 (1.9%)	All for local wound revision or hematoma evacuation
Readmission within 30 days	3 (1.4%)	Secondary wound care or infection
Follow-up duration (months)	10.4 ± 3.8	No locoregional recurrences during follow-up

Baseline demographic, clinical, and surgical characteristics of patients undergoing mastectomy with or without immediate reconstruction. Data are presented as mean ± SD for continuous variables and as frequency (percentage) for categorical variables. Abbreviations: ASA—American Society of Anesthesiologists; BMI—Body Mass Index; SLNB—Sentinel Lymph Node Biopsy; ALND—Axillary Lymph Node Dissection; IBR—Immediate Breast Reconstruction.

**Table 2:** Summary of reconstructive techniques performed among patients who underwent immediate breast reconstruction (IBR).

Reconstructive Technique	Number (*n*)	Percentage (%)	Notes
Direct-to-Implant Reconstruction	42	48.8%	Most common technique; selected in patients with favorable skin envelopes and minimal comorbidities.
Two-Stage Expander–Implant Reconstruction	34	39.5%	Used when skin quality required progressive expansion.
Autologous Reconstruction (Latissimus Dorsi Flap)	10	11.6%	Limited use due to increased operative time and resource constraints.
Total	86	100%	—

**Table 3:** Distribution of Immediate Reconstruction by HER2 Status.

HER2 Status	No Reconstruction	Immediate Reconstruction	% Immediate
Negative	67	47	41.2%
Positive	17	12	41.4%

Distribution of patients undergoing immediate breast reconstruction (IBR) according to HER2 receptor status. Values are presented as absolute numbers and percentages. Abbreviations: HER2—Human Epidermal Growth Factor Receptor 2; IBR—Immediate Breast Reconstruction.

**Table 4:** Logistic Regression Predicting Immediate Breast Reconstruction from HER2 Status and Clinical Covariates.

Predictor	B	SE	Wald z	*p*	OR	95% CI OR
Intercept	9.17	3.02	3.04	0.002	-	-
HER2+	0.06	0.70	0.08	0.933	1.06	[0.30, 3.79]
Age	−0.14	0.04	−3.44	<0.001	0.87	[0.80, 0.93]
BMI	0.10	0.06	1.61	0.107	1.11	[0.98, 1.26]
Smoking	−0.27	0.76	−0.35	0.724	0.77	[0.17, 3.47]
Comorbidities	−0.85	0.68	−1.25	0.211	0.43	[0.11, 1.64]
Ki-67 (%)	−0.00	0.02	−0.28	0.782	1.00	[0.97, 1.03]
CHT neoadjuvant	−1.20	0.72	−1.67	0.095	0.30	[0.08, 1.09]
pT	−0.24	0.22	−1.10	0.270	0.79	[0.51, 1.25]
pN	−0.35	0.21	−1.71	0.087	0.70	[0.45, 1.08]
Surgery (main)	−1.49	0.48	−3.13	0.002	0.23	[0.09, 0.59]

Proportion of patients receiving immediate breast reconstruction (IBR) across quartiles of the Ki-67 proliferation index. Ki-67 values are expressed as percentages. No significant trend was observed across quartiles (*p* = 0.58). Abbreviations: Ki-67—Tumor proliferation index; IBR—Immediate Breast Reconstruction.

**Table 5:** Rates of Immediate Reconstruction Across Ki-67 Quartiles.

Ki-67 Quartile	*n*	Mean Ki-67 (%)	% Immediate Reconstruction
1 (lowest)	32	5.88	50.0%
2	32	15.2	37.5%
3	32	25.3	34.4%
4 (highest)	32	53.6	37.5%

Multivariate logistic regression model evaluating predictors of immediate breast reconstruction (IBR), including tumor biology (HER2 status and Ki-67 index) and clinical covariates. Odds ratios (OR) with 95% confidence intervals (CI) are shown. Significant predictors were age (younger patients more likely to undergo IBR) and type of surgery (less extensive procedures associated with higher IBR rates). Abbreviations: HER2—Human Epidermal Growth Factor Receptor 2; Ki-67—Tumor proliferation index; OR—Odds Ratio; CI—Confidence Interval; BMI—Body Mass Index; CHT—Chemotherapy; pT—Pathologic Tumor Size; pN—Pathologic Nodal Stage; IBR—Immediate Breast Reconstruction.

**Table 6:** Logistic Regression Predicting Immediate Breast Reconstruction from Triple-Negative Breast Cancer Status and Clinical Covariates.

Predictor	B	SE	Wald z	*p*	OR	95% CI OR
Intercept	5.94	2.06	2.88	0.004	—	—
TNBC (1 = yes)	−0.82	0.82	−1.00	0.317	0.44	[0.08, 2.18]
Age	−0.09	0.03	−3.10	0.002	0.91	[0.86, 0.96]
BMI	0.06	0.05	1.11	0.268	1.06	[0.96, 1.18]
Smoking	−0.43	0.61	−0.71	0.480	0.65	[0.19, 2.13]
Comorbidities	−1.19	0.61	−1.95	0.052	0.30	[0.09, 1.00]
CHT neoadjuvant	−0.55	0.56	−0.99	0.323	0.58	[0.18, 1.67]
pT	−0.11	0.15	−0.73	0.468	0.90	[0.68, 1.20]
pN	0.02	0.12	0.17	0.868	1.02	[0.79, 1.30]
Surgery (main)	−0.80	0.28	−2.91	0.004	0.45	[0.25, 0.74]

Multivariate logistic regression assessing the influence of triple-negative breast cancer (TNBC) status and clinical covariates on immediate breast reconstruction (IBR). Results are expressed as odds ratios (OR) with 95% confidence intervals (CI). TNBC status was not a significant predictor; age and surgical extent remained the main determinants. Abbreviations: TNBC—Triple-Negative Breast Cancer; OR—Odds Ratio; CI—Confidence Interval; BMI—Body Mass Index; CHT—Chemotherapy; pT—Pathologic Tumor Size; pN—Pathologic Nodal Stage; IBR—Immediate Breast Reconstruction.

## Data Availability

Data supporting the findings of this study are available upon reasonable request from the corresponding authors. Due to legal constraints and institutional policies, the dataset cannot be made publicly accessible.

## Abbreviations

The following abbreviations are used in this manuscript:

ALND	Axillary Lymph Node Dissection
BMI	Body mass index
BRCA	Breast Cancer Gene (BRCA1/BRCA2)
CHT	Chemotherapy
CI	Confidence Interval
CT	Computed Tomography
DCIS	Ductal Carcinoma in Situ
ECIS	European Cancer Information System
ER	Estrogen Receptor
ESMO	European Society of Breast Cancer Specialists
H&E	Hematoxylin and Eosin
HER2	Human Epidermal Growth Factor Receptor 2
HR	Hormone Receptor
IBR	Immediate Breast Reconstruction
IHC	Immunohistochemistry
IRB	Institutional Review Board
MRI	Magnetic Resonance Imaging
NCCN	National Comprehensive Cancer Network
NSM	Nipple-Sparing Mastectomy
OR	Odds Ratio
PET-CT	Positron Emission Tomography-Computed Tomography
PR	Progesterone Receptor
pT/pN	Pathologic Tumor size/Pathologic Nodal Stage
SD	Standard Deviation
SLNB	Sentinel Lymph Node Biopsy
SSM	Skin-Sparing Mastectomy
STROBE	Strengthening the Reporting of Observational Studies in Epidemiology
TNBC	Triple-Negative Breast Cancer
